# Supplementary material for: Anti-Salmonella Defence and Intestinal Homeostatic Maintenance In Vitro of a Consortium Containing Limosilactobacillus fermentum 3872 and Ligilactobacillus salivarius 7247 Strains in Human, Porcine, and Chicken Enterocytes
Source: Antibiotics (Basel). 2023 Dec 28;13(1):30. doi: 10.3390/antibiotics13010030 (PMC10812507; doi:10.3390/antibiotics13010030)
Supplement: Supplementary file 1 [file antibiotics-13-00030-s001.zip › antibiotics-2734039-supplementary.pdf]

Supplementary Materials

Table S1. Primers used for qRT-PCR analysis of SE genes responsible for the intestinal colonization and virulence.

**Genes**	Function	**Primer**	**Sequence**
sopB	Adherence	Forward	GCGTCAATTTCATGGGCTAAC
		Reverse	GGCGGCGAACCCTATAAACT
invH	Invasion	Forward	CCCTTCCTCCGTGAGCAAA
		Reverse	TGGCCAGTTGCTCTTTCTGA
sipB	Type 3 secretion system	Forward	GCCACTGCTGAATCTGATCCA
		Reverse	CGAGGCGCTTGCTGATTT
pipB	Type 3 secretion system	Forward	GCTCCTGTTAATGATTTCGCTAAAG
		Reverse	GCTCAGACTTAACTGACACCAAACTAA
orf245	Type 3 secretion system	Forward	CAGGGTAATATCGATGTGGACTACA
		Reverse	GCGGTATGTGGAAAACGAGTTT
sipA	Type 3 secretion system	Forward	CAGGGAACGGTGTGGAGGTA
		Reverse	AGACGTTTTTGGGTGTGATACGT
ssaV	Type 3 secretion system	Forward	GCGCGATACGGACATATTCTG
		Reverse	TGGGCGCCACGTGAA
spvB	Survival in macrophages	Forward	TGGGTGGGCAACAGCAA
		Reverse	GCAGGATGCCGTTACTGTCA
mgtC	Survival in macrophages	Forward	CGAACCTCGCTTTCATCTTCTT
		Reverse	CCGCCGAGGGAGAAAAAC
sodC	Survival in macrophages	Forward	CACATGGATCATGAGCGCTTT
		Reverse	CTGCGCCGCGTCTGA
tatA	Cell wall and cell membrane integrity	Forward	AGTATTTGGCAGTTGTTGATTGTTG-3^{r}
		Reverse	ACCGATGGAACCGAGTTTTTT-3^{r}
hflK	Cell wall and cell membrane integrity	Forward	AGCGCGGCGTTGTGA
		Reverse	TCAGACCTGGCTCTACCAGATG
ompR	Cell wall and cell membrane integrity	Forward	TGTGCCGGATCTTCTTCCA
		Reverse	CTCCATCGACGTCCAGATCTC
mrr1	Efflux pump regulator	Forward	CCATCGCTTCCAGCAACTG
		Reverse	TCTCTACCATGAACCCGTACAAATT
lrp	Virulence regulation	Forward	TTAATGCCGCCGTGCAA
		Reverse	GCCGGAAACCAAATGACACT
xthA	Exo/endonuclease activity	Forward	CGCCCGTCCCCATCA
		Reverse	CACATCGGGCTGGTGTTTT
rpoS	Oxidative stress	Forward	TTTTTCATCGGCCAGGATGT
		Reverse	5^{r}-CGCTGGGCGGTGATTC
ssrA	Metabolism	Forward	CGAGTATGGCTGGATCAAAACA
		Reverse	TGTACGTATTTTTTGCGGGATGT
rfbH	Lipopolysaccharide biosynthesis	Forward	ACGGTCGGTATTTGTCAACTCA
	Function	Reverse	TCGCCAACCGTATTTTGCTAA

Table S**2**. Human, porcine and chicken primers used for qRT-PCR analysis of host immune response.

Primer	Sense primer	Antisense primer	Reference
Human TLR4	TGCACAGGACAGAACATCTCTGGA	AGCTCCTGCAGGGTATTCAAGTGT	[106]
Human TLR9	GTGCCCCACTTCTCCATG	GGCACAGTCATGATGTTGTTG	[107]
Human IAP	CATACCTGGCTCTGTCCAAGA	CGCTCCACCAACTAAGAACG	[108]
Human β- actin	TCACCCACACTGTGCCCATCTACGA	CAGCGGAACCGCTCATTGCCAATG	[106]
Porsine TLR4	CTCTGCCTTCACTACAGAGA	CTGAGTCGTCTCCAGAAGAT	[109]
Porsine TLR9	GTGGAACTGTTTTGGCATC	CACAGCACTCTGAGCTTTGT	[109]
Porsine β- actin	CATCACCATCGGCAACGA	GCGTAGAGGTCCTTCCTGATGT	[109]
Chicken TLR4	TGCACAGGACAGAACATCTCTGGA	AGCTCCTGCAGGGTATTCAAGTGT	[60]
Chicken TLR21	AGAAGGTGTCGGAGGATGGTG	GGGCTCCAAATGCTGACTGC	[60]
Chicken β-actin	ACGTCTCACTGGATTTCGAGCAGG	ACGTCTCACTGGATTTCGAGCAGG	[60]
